# Experimental study of platelet-rich fibrin affecting the healing of seawater-impregnated wounds in rabbits

**DOI:** 10.3389/fcell.2025.1695908

**Published:** 2026-02-26

**Authors:** Ke Xie, Zeyu Dai, Daiqing Chen, Xiaojing Peng, Yipu Lu, Jiansheng Zheng

**Affiliations:** 1 Department of Burns and Plastic Surgery, The 909th Hospital, School of Medicine, Xiamen University, Zhangzhou, Fujian, China; 2 Department of Plastic Surgery, Xiamen Hospital of Traditional Chinese Medicine, Xiamen, Fujian, China

**Keywords:** platelet-rich fibrin (PRF), wound healing, seawater immersion, angiogenesis, rabbit

## Abstract

**Background:**

To investigate the effect of platelet-rich-fibrin (PRF) on the healing of seawater-impregnated wounds in rabbits and to explore the mechanism.

**Material and Methods:**

Twenty-four New Zealand White rabbits were used. Two full-thickness skin wound models were created on each rabbit’s back and immersed in seawater. According to a random number table, one wound on each rabbit was assigned to the PRF group (treated with PRF gel) and the contralateral wound served as the control (untreated). Wound healing rate, histomorphology, bacteriology, and neovascularization (via CD34 immunohistochemistry) were assessed on days 1, 4, 7, and 14 post-operation. Statistical analysis was performed using ANOVA with paired samples t-test and Bonferroni correction.

**Results:**

Wounds in the control group exhibited significant erythema, edema, and inflammatory exudate, with a healing rate of only 53.5% ± 3.2% by day 14. In contrast, PRF-treated wounds showed minimal signs of infection, reduced inflammation, and were almost completely healed (92.9% ± 0.9%) by day 14. The wound healing rate was significantly higher in the PRF group at all time points (P < 0.01). Bacteriological analysis identified BacAutologous PRF significantly promotes the healing of seawater-immersed wounds in rabbits. The mechanism is likely multifactorial, involving the promotion of angiogenesis, reduction of inflammation, and potential inhibition of bacterial growth. PRF represents a promising therapeutic option for the management of seawater immersion wounds. d formation of new capillaries and fibroblasts. Immunohistochemistry confirmed a significantly higher density of CD34^+^ neovessels in the PRF group at days 4, 7, and 14 (P < 0.05).

**Conclusion:**

Autologous PRF significantly promotes the healing of seawater-immersed wounds in rabbits. The mechanism is likely multifactorial, involving the promotion of angiogenesis, reduction of inflammation, and potential inhibition of bacterial growth. PRF represents a promising therapeutic option for the management of seawater immersion wounds.

## 1 Background

In the 21st century, maritime land, marine resources, maritime rights and maritime security cannot be ignored. With the increasing frequency of maritime activities, seafarers in a variety of accidental injuries to the sea is very easy to happen, maritime medical rescue has become an important subject of aviation and maritime medicine in the world. Maritime activities are increasing globally, and with them, the risk of accidental injuries at sea. Consequently, maritime medical rescue has become a critical focus in aviation and maritime medicine. Due to the characteristics of seawater low temperature, high sodium, high osmosis and rich in microorganisms, seawater immersion injuries are more complex and serious than ordinary land injuries. Seawater immersion complicates wound healing due to its low temperature, high salinity, hyperosmolarity, and microbial richness, making such injuries more severe than those sustained on land. For the traumatic surface, after seawater immersion, the local inflammatory reaction is aggravated, and the degenerative and necrotic tissues increase; the cell repair and proliferation function is inhibited, and the granulation tissue growth and maturation are slow; the myofibroblast production is too little. Factors such as hypothermia, disturbance of the normal metabolic level of the body, systemic infections and decreased resistance are also important factors affecting the healing of seawater-immersion wounds (Eming et al., 2009; Wang et al., 2016)^.^ Platelet-rich fibrin, first reported by the French scholar Choukroun in 2001 (Dohan et al., 2006), is a second generation platelet concentrate (Mansilla et al., 2005), which is a platelet-rich fibrin clot obtained by primary centrifugation of autologous venous blood without the addition of anticoagulants and possesses abundant cell growth factors, thus promoting cell proliferation, microvascular neovascularization, etc., with good tissue It has good tissue repair and regenerative effects. Platelet-rich fibrin (PRF), a second-generation platelet concentrate first described by Choukroun et al. (Dohan et al., 2006), is an autologous fibrin matrix containing platelets, leukocytes, and a rich reservoir of growth factors. It promotes cell proliferation and angiogenesis, demonstrating excellent tissue repair and regenerative properties (Mansilla et al., 2005). Therefore, it has received more attention in oral and maxillofacial surgery, orthopedics, plastic surgery, and sports medicine. In this study, we intend to elucidate the mechanism of action of PRF, evaluate its use value in clinical management, improve the treatment level of seawater-impregnated wounds, and lay the theoretical foundation for future clinical treatment through the experimental study of PRF on seawater-impregnated wounds in New Zealand rabbits. This study aims to investigate the efficacy and potential mechanism of PRF in promoting the healing of seawater-impregnated wounds in a rabbit model, to evaluate its clinical potential, and to improve treatment strategies for these challenging injuries.

## 2 Materials and methods

### 2.1 Experimental animals

24 Westland white rabbits, male and female, 2.5–3 kg. Provided by Songlian Experimental Animal Farm, Songjiang District, Shanghai, China, provided and fostered by the Animal Experiment Center of Southeast Hospital, Xiamen University, with normal diet. Certificate of Conformity No.: 20170008003061. This study was approved by the Ethics Committee of Southeast Hospital, Xiamen University (Grant No: 2019-002-01). All animal experiments met the requirements of the ethics committee. Twenty-four New Zealand White rabbits (2.5–3 kg, male and female) were provided by the Songlian Experimental Animal Farm (Shanghai, China) and housed at the Animal Experiment Center of Southeast Hospital, Xiamen University. The study was approved by the Institutional Ethics Committee (Grant No: 2019-002-01).

### 2.2 Animal group

Twenty-four New Zealand White rabbits were selected, and two seawater-impregnated trauma models were established on the left and right side of each rabbit’s spine, with symmetry on both sides. The wounds on one side of each rabbit were randomly assigned to the PRF group using a random number table, with the contralateral wounds serving as the control group.

### 2.3 Main reagents and instruments

Hematoxylin staining solution: Fuzhou Maixin Biotechnology Co., Ltd., 0.5% eosin ethanol solution: Fuzhou Maixin Biotechnology Co., Ltd., sodium citrate buffer; fuzhou Maxin Biotechnology Co., Ltd., filter 0.22 um filter: Millipore, USA, PV-9004 immunohistochemical detection kit: Beijing Zhongshan Golden Bridge Biotechnology Co., Ltd., ready-to-use mouse anti-human CD34 monoclonal antibody, ready-to-use horseradish peroxidase-labeled goat anti-mouse IgG polyclonal antibody: Fuzhou Maxin Biotechnology Co., Ltd. HEAEUS CRYOFUHE 6000i low-temperature large-capacity centrifuge (Beijing Dongxun Tiandi Medical Instrument Co., Ltd.) IXUS210 digital camera was purchased from Canon, Japan, WI93961 medical image analysis system was purchased from Beijing Beiruda Pharmaceutical Technology Co., Ltd., DM4B biological microscope was purchased from Leica, Germany. Key reagents and instruments included hematoxylin-eosin staining solutions (Maixin, China), mouse anti-human CD34 monoclonal antibody (Maixin, China), an immunohistochemical detection kit (Zhongshan Jinqiao, China), a CRYOFUGE 6000i centrifuge (Dongxun Tiandi, China), and a DM4B biological microscope (Leica, Germany).

### 2.4 Seawater and PRF preparation

**FIGURE 1:**
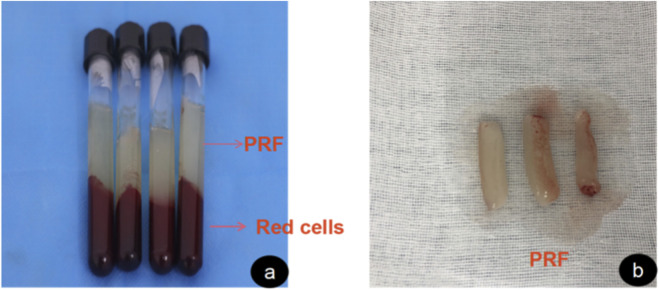
Platelet-rich fibrin preparation process. **(a)** shows the appearance of whole blood after centrifugation. **(b)** shows the extracted intermediate layer of platelet-rich fibrin.

Seawater was taken from a sea area in Zhangzhou, Fujian. The preparation of PRF was in accordance with the principle of ‘freshly made’. 1 hour before the experiment, 15 mL blood was collected by rabbit heart blood collection method, placed in a sterile and additive-free centrifuge tube, and immediately centrifuged at 3,000r/min (1,408 × g) for 10 min. After centrifugation, the liquid in the visible tube was divided into 3 parts, and the top was light yellow clear liquid, i.e., platelet-poor plasma layer; the middle is white gel, which is a platelet-rich fibrin gel; the bottom is dark red jelly, namely red blood cells and cell debris. The supernatant was discarded, and the white clot in the middle was carefully removed and placed on a dry sterile medical gauze pad. After the impurities and red blood cell debris at the bottom were removed by ophthalmic scissors and toothless tweezers, the PRF membrane was made by gently pressing with sterile gauze for 10s (Figure 1).

### 2.5 Establishment of a seawater immersion trauma model in rabbits

New Zealand rabbits were fixed in the prone position after anesthesia, disinfected by shaving the back, disinfected with iodophor, and laid on a towel, and two square traumas with 2 cm sides of total skin defect were cut at 1.5 cm on each side of the rabbit’s spine, deep to the fleshy membrane layer (Liu et al., 2020). Immediately after injury, the rabbits were tied and fixed on an iron frame with their heads exposed to the sea and immersed in seawater for 60 min, and then disinfected with iodine volt after drying the seawater on the body surface with gauze. The prepared PRF gel was then applied to the wounded surface of the PRF group and wrapped with sterile gauze to return to the cage, while the control group was disinfected directly and wrapped with sterile gauze to return to the cage.

### 2.6 Observation index and detection method

#### 2.6.1 Wound healing

Six rabbits in each group were taken on postoperative days 1, 4, 7, and 14, respectively, to observe and record the general healing of the wounds. A digital camera was also used to take pictures of all the wounds, compare the size of the wounds, and measure the unhealed area of the wounds by Image-Pro Plus 6.0 software to calculate the healing rate of the wounds. The wound healing rate = (initial wound area - unhealed area)/initial wound area × 100%.

#### 2.6.2 Histomorphology

The rabbits were anesthetized at 1, 4, 7, 14 days postoperatively, respectively, and the traumatic surface and peri-traumatic 5-mm tissue were excised. The tissues were immediately immersed in 10% paraformaldehyde for fixation, routinely paraffin-embedded, sectioned (thickness of 5 μm), HE stained, and the histopathological changes of the trauma were observed under a 100× light microscope.

#### 2.6.3 Bacteriological detection

Traumatic tissues were obtained by the same method as above, soaked in liquid nitrogen, and muscle tissues were ground into powder with a tissue grinder (Tissuelyser-48, NetSense Technology) under aseptic conditions, which were added to 6 mL of PBS solution with vortex shaking for 2 min, centrifuged at 10,000×g for 5 min, and the supernatant was taken and diluted step by step in a 10-fold concentration gradient. Part of the supernatant was used for bacterial culture, respectively, for bacterial identification, and part of the supernatant was dropped onto an agar plate and spread evenly, and incubated at 37 °C for 24 h to observe the formation of bacterial colonies.

#### 2.6.4 Neovascularization status

It’s detected by immunohistochemical diaminobenzidine staining. Tissues were routinely dewaxed, gradient transparent, 3% H2O2 inactivated to block endogenous nucleic acid endonucleases, and 5% BSA closed. CD34 primary antibody at a concentration of 1:1,000 was added dropwise, refrigerated overnight at 4 °C, secondary antibody at a concentration of 1:200 was added dropwise, incubated for 30 min at 37 °C, DAB color development, hematoxylin re-staining, and neutral gum sealing. Positive proteins were identified as those showing brownish-yellow particles in the cytoplasm. Images were acquired with Image-Pro Plus 6.0 software, and three fields of view were selected for each section at the same site under 100× light microscope to compare the average number of positive cells in each group.

### 2.7 Statistical analysis

SPSS 22.0 software was used to analyze the experimental data, and all data were normally distributed and expressed in terms of overall comparisons between groups by analysis of variance (ANOVA) with paired sample t-test and Bonferroni correction. P < 0.05 was considered statistically significant. The sample size was determined based on previous similar studies and preliminary experiments to ensure adequate power. Exact P-values are reported.

## 3 Experimental results

### 3.1 Bacteriological evaluation of the trauma surface

**FIGURE 2:**
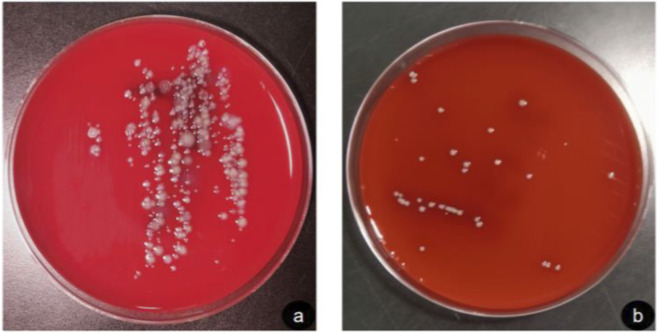
Bacteriological assessment of seawater immersion trauma **(a)** control group **(b)** experimental group.

Results of bacteriological assessment: day 4 trauma bacterial cultures were taken and the identification results suggested that the bacteria cultured in the 2 seawater soaked trauma groups were of the genus *Bacillus*. The results of the agar plate bacterial culture are shown in Figure 2 A shows the seawater soaked trauma uncovered PRF group with a large number of bacterial colonies forming on the agar plate and dense bacterial growth. B shows the seawater soaked trauma filled with PRF group with a small number of bacterial colonies forming on the agar plate. PRF plays an important role in this effect. In contrast, in earlier reports, seawater contained a large number of microorganisms, especially Gram-negative bacteria. Apparently, the results of the present experiment are consistent with the previous findings (Ma et al., 2017). This qualitative assessment demonstrated a visible reduction in bacterial colony formation in the PRF-treated wounds compared to the control.

### 3.2 Gross trauma observation

**FIGURE 3:**
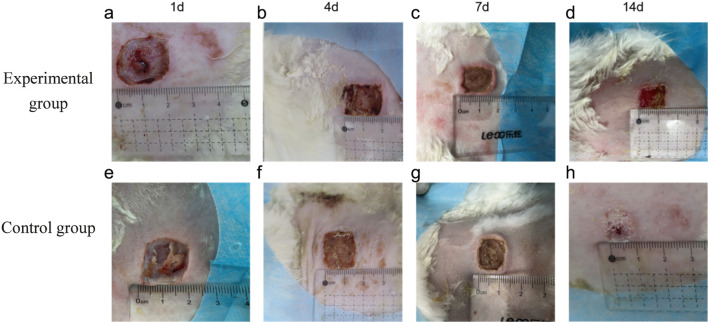
General wound healing at each time point in the experimental and control groups. **(a–d)** represent the appearance of the wounds in the experimental group on Day 1, Day 4, Day 7, and Day 14, respectively. **(e–h)** represent the appearance of the wounds in the control group on Day 1, Day 4, Day 7, and Day 14, respectively.

The rabbits in each group tolerated well postoperatively. In this experiment, the wound healing of each group was observed and recorded before taking the material on days 1, 4, 7 and 14. On day 1 after the injury, the PRF group had a relatively clean wound, and the PRF gel was also visible to the naked eye as a tight fit to the wound, and the wound was dry, with no bleeding or inflammatory secretions. In the control group, there was obvious inflammatory exudation, a small amount of surrounding secretion, and the wound surface was red, and the skin temperature was slightly higher than the surrounding tissues when touched (Figures 3a,b); 4 days after the operation, the wound surface of the PRF group was covered with thin scabs, the wound surface was red and moist, and the PRF was basically integrated into the wound surface, the surrounding area was clean, and no bleeding or inflammatory secretions were seen. In the control group, the color of the trauma was dark red, a little exudation was still visible, and there was a small amount of bloody crust around the trauma (Figures 3c,d); on the 7th day after surgery, the color around the trauma in the PRF group was close to the skin, the scab had thickened and completely covered the trauma, the appearance of the trauma was dry, the PRF gel was completely absorbed, the trauma was clean, no exudation, the temperature was close to the surrounding area when touched, and the texture was soft and elastic. In the control group, obvious redness and swelling were still visible around the trauma, with a redder color than the surrounding skin and a slightly higher skin temperature than the surrounding skin. (Figures 3e,f); on the 14th postoperative day, the scab of the PRF group had fallen off and the wound had basically healed, with the area reduced by 80% compared with the previous one, the surrounding area was dry and clean, the texture was soft and elastic, the temperature was consistent with the surrounding temperature, and the surrounding hair was covered, reaching the standard of complete healing. In the control group, the trauma surface was dark red in color and obviously different from the surrounding skin, the trauma surface area was reduced by less than 50%, inflammatory exudation was visible, and the temperature was higher than the surrounding area. The overall observation was that the control group did not grow as well as the PRF group (Figures 3g,h).

### 3.3 Wound healing rate

On postoperative day 4, the wound healing rate was 30.5% ± 2.5% in the PRF group of rabbits; 20.9% ± 2.6% in the control group. At day 7, the wound healing rate was 52.1% ± 1.8% in the PRF group and 36.0% ± 3.3% in the control group. At day 14, the trauma surface was basically healed and the trauma surface color was close to normal skin with a healing rate of 92.9% ± 0.9% in the PRF group and 53.5% ± 3.2% in the control group, and inflammatory reactions were still visible. The trauma surface healing rate was significantly higher in the PRF group than in the control group at all time points, and the differences were statistically significant (P < 0.01) (Table 1). It was concluded that PRF, as a treatment with convenient extraction, has good efficacy in repairing wounds and has a certain anti-infective effect, which can accelerate the healing of seawater-impregnated wounds.

**TABLE 1:** Wound healing rate (%) in experimental group and control groups in different days.

Group	Samples	1 day	4 days	7 days	14 days
Experimental group	24	0.0 ± 0.0	30.5 ± 2.5	52.1 ± 1.8	92.9 ± 0.9
Control group	24	0.0 ± 0.0	20.9 ± 2.6	36.0 ± 3.3	53.5 ± 3.2
*t*		—	5.53	11.59	26.76
*P*		—	0.003	<0.001	<0.001

The number of samples at each time point in each group is 6, using paired sample t-test; main effect of treatment factor, F = 668.57, P < 0.001; main effect of time factor, F = 2391.87, P < 0.001, interaction effect of treatment and time, F = 176.55, P < 0.001.

### 3.4 Histomorphological observation

**FIGURE 4:**
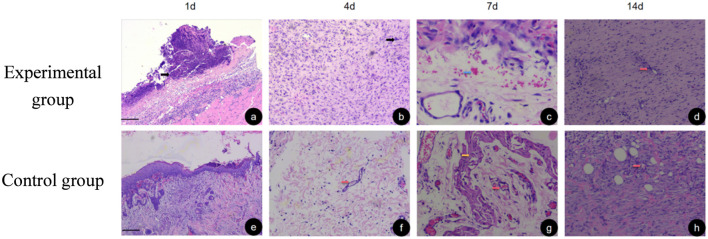
HE staining at HE 100 scale = 50 um at different time points, neutrophils indicated by black arrows, neovascularization indicated in red arrows, bleeding lesions indicated in green arrows, and collagen fibers indicated by yellow arrows. **(a–d)** show the hematoxylin and eosin (HE) staining results of the wound tissues from the experimental group on Day 1, Day 4, Day 7, and Day 14, respectively. **(e–h)** show the HE staining results of the wound tissues from the control group on Day 1, Day 4, Day 7, and Day 14, respectively.

The density of neovascularization gradually increased with time on the experimental side and control side. The increase in neovascular density was significant in the 7-day group compared with the 3-day group and in the 14-day group compared with the 7-day group, respectively. At 1 d postoperatively, more inflammatory cell infiltration was seen in both the PRF and control groups, with more pronounced in the control group. At 3 d postoperatively, inflammatory cell infiltration was observed at the base of the wound in both the control and PRF groups, but a small amount of neovascularization was present in the PRF group, and the wall of the neovascularization was thinner. At 7 d after surgery, the basal fibrin proliferation was active in the PRF group, and the number of neovascularization was significantly higher than that in the control group, and the wall of neovascularization was gradually differentiated and matured. At 14 d postoperatively, the number of neovascularization in the PRF group increased significantly, while the number of vessels on the trabecular surface of the control group was relatively small and the PRF group was rich in vessels (As shown in Figure 4). Histological evaluation revealed that the PRF group consistently exhibited less inflammatory cell infiltration and more robust neovascularization and fibroblast proliferation compared to the control group at all time points (Figure 4).

### 3.5 CD34 neovascularization

**FIGURE 5:**
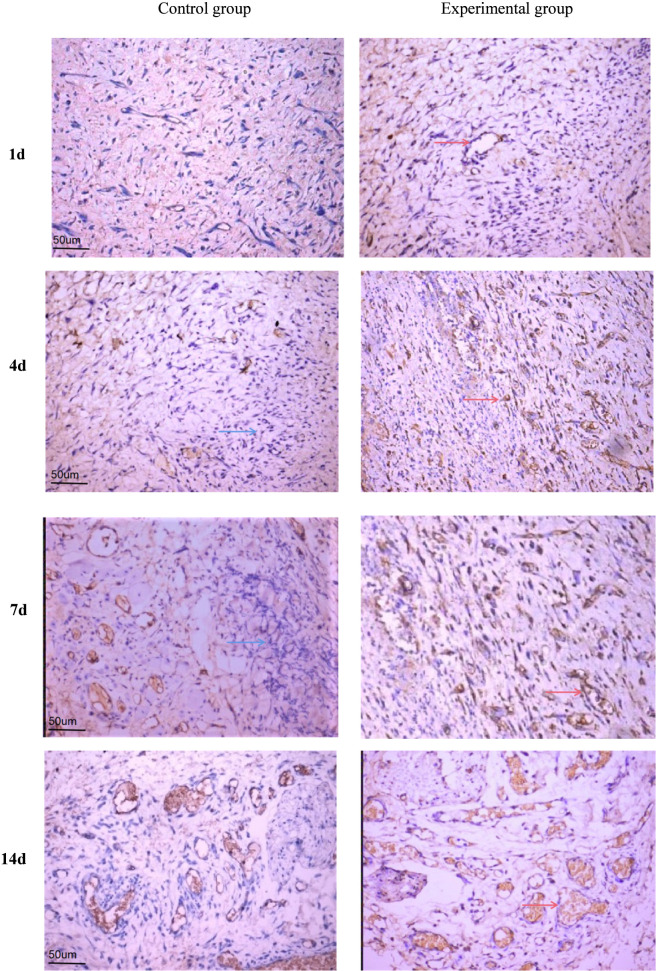
Immunohistochemistry of CD34 in the control group and the experimental group at each time point, scale = 50 um, red arrow: neovascularization, and blue arrow: inflammatory cells.

**FIGURE 6 F6:**
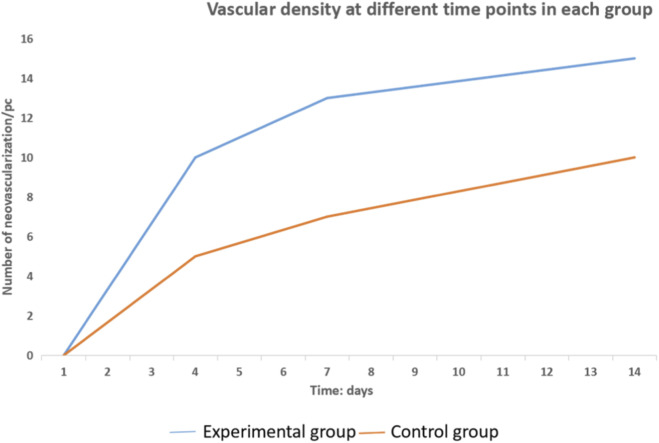
Vascular growth trend diagram between experimental and control groups at different time points.

From 1 to 14 days after surgery, the neovascularization in the PRF group gradually increased and was significantly higher than that in the control group, and the results of statistical analysis are shown in (Figure 5,6, [Fig F6]; Table 2): the results of paired t-test showed that the neovascularization density in the experimental and control groups at 4, 7 and 14 days were statistically different (P < 0.05). Histological evaluation revealed that the PRF group consistently exhibited less inflammatory cell infiltration and more robust neovascularization and fibroblast proliferation compared to the control group at all time points (Figure 4).

**TABLE 2:** Neovascular density (individual/HP, in experimental and control groups, ± S).

Group	Samples	1d	4d	7d	14d
Experimental group	24	0.0 ± 0.0	3.8 ± 0.8	8.2 ± 1.2	11.5 ± 1.0
Control group	24	0.0 ± 0.0	2.2 ± 0.8	6.0 ± 1.3	9.0 ± 0.9
*t*		—	3.371	4.54	5.839
*P*		—	0.020	0.006	0.002

The number of samples at each time point is 6, using the t-test of paired samples; main effect of treatment factor F = 30.08, P < 0.001; main effect of time factor F = 243.50, P < 0.001, the interaction effect of treatment and time, F = 3.69, P = 0.005.

## 4 Discussion

### 4.1 Hazards of seawater-immersed wounds and research status

In recent years, the marine industry has been growing and marine workers are vulnerable to various open injuries (Diaz, 2014). It has been shown that seawater immersion leads to the release of large amounts of inflammatory factors (e.g., IL-8, TNF, and NO) from the injured area, resulting in multiple organ damage in the body and increasing the likelihood of diffuse intravascular coagulation, which ultimately leads to increased mortality (Ma et al., 2017; Yan et al., 2016; Yuan et al., 2018; Stacy et al., 2014; Ji et al., 2016; Ai et al., 2014). In addition, seawater is a complex hypertonic alkaline solution whose chemical composition is mainly NaCl, but also includes KCl, CaCl2, MgCl2, and MgSO4 in different proportions. When skin wounds are immersed in seawater for a long time, they are more prone to tissue necrosis and infection, prolonging the healing time of skin wounds and leading to chronic wounds. However, there are few reports on the effects of seawater immersion on total skin wounds and their occurrence mechanisms. In contrast, in this experiment, we established a seawater immersion wound model and observed a significant delay in healing efficiency of seawater immersion wounds. And currently, for seawater immersion wounds, in terms of treatment, it is mainly routine debridement and drug exchange and anti-inflammation, and it has been reported in the literature that alkaline fibroblast growth factor and hepatocyte growth factor recombinant plasmid have achieved some efficacy in promoting wound healing (Ning et al., 2009; Zhu et al., 2025; Ning et al., 2010; Zhang et al., 2012).

### 4.2 Characteristics of PRF and its advantages in wound healing

Platelet rich plasma (PRP), as the first generation platelet concentrate, has been proven to promote wound repair by promoting basal vascularization of the wound (Zheng et al., 2021). It promotes angiogenesis, collagen synthesis, and epithelialization by releasing various growth factors such as PDGF, VEGF, TGF-β, etc., significantly accelerating wound healing. However, the preparation of PRP requires the addition of exogenous thrombin, which has limitations such as poor stability and transient release of growth factors. PRF is a second-generation platelet concentrate, rich in fibrin, leukocytes, and a large number of growth factors. Compared with the first generation, it is easier to make, its biological properties are more stable and durable, and no allogeneic materials such as thrombin are added, which not only makes the preparation easier but also well avoids safety issues such as allergic reactions, immune rejection, and cross-infection. When activated, PRF slowly releases a variety of cytokines, such as transforming growth factor-β, platelet-derived growth factor and vascular endothelial growth factor (Dohan Ehrenfest et al., 2018)^.^ The combined action of multiple growth factors can improve soft tissue repair and reduce inflammatory response and edema (Strauss et al., 2020) On the other hand, the release of some antimicrobial active peptides in leukocytes, platelets themselves and platelet activation to resist microorganisms can prevent trauma infection (Drago et al., 2013). And fibrin can prevent the loss of growth factors, leukocytes and other active substances in the wound surface, prolong the local effect time of PRF, so that the tissue has a relatively long-lasting source of growth factors, which can further promote wound healing.

### 4.3 Optimization of PRF preparation methods

Currently, there are different methods of preparing PRF, with no fixed reference standard and mostly a summary of experience. It was found that the amount of transforming growth factor-β and platelet-derived growth factor-AB released was significantly higher in the preparation by centrifugation at different speeds, 3,000 rpm for 10 min, respectively, than in the other six groups (Li et al., 2012). Therefore, in this experiment, the experimental method of centrifugation at 3,000 rpm for 10 min was used, and during the study, several preparation experiences revealed that arterial blood was easier to centrifuge successfully than venous blood after centrifugation of the same amount of whole blood. To summarize the possible reasons: after the collection of whole blood without the addition of anticoagulants, a large number of platelets are rapidly activated, while the oxygen content in arterial blood is higher at this time, so the activity of the corresponding thrombin is relatively more active. PRF gel and PRF film are observed under electron microscopy, both of them gather a large number of platelets and leukocytes, and there is no other difference, the PRF film fibrin network is more dense (Xuan et al., 2015). And there are studies during which different centrifugation times and rotational speeds of whole blood were found to result in different formation results (Li et al., 2019). In this study, one variable was kept constant, and as the rotational speed and centrifugal force increased, the centrifugation time was prolonged and platelets were destroyed, and the destruction of other components of the blood increased accordingly, in agreement with the results.

### 4.4 Therapeutic effects of PRF on seawater-immersed wounds

In this study, after applying PRF to seawater-soaked wounds, the group found that the PRF group showed less inflammatory response and significant proliferation of new capillaries and fibroblasts than the control group. The mechanism of PRF release of vascular endothelial growth factor (VEGF), which promotes the formation of neovascularization and thus rapid vascularization of the wounds, and the combined effect of leukocytes and platelets to reduce the inflammatory response and, to some extent, the infection, were suggested. There are some shortcomings in this study, as VEGF-related indicators were not added, and the expression of various growth factors contained in PRF was not quantitatively analyzed, and our group will further improve the study of the mechanism in the follow-up study. Our results demonstrate that autologous PRF significantly accelerates the healing of seawater-immersed wounds. The promoted healing is likely mediated through the known capacity of PRF to serve as a sustained-release scaffold for key growth factors like VEGF, PDGF, and TGF-β, which collectively stimulate angiogenesis and tissue regeneration (Dohan Ehrenfest et al., 2018; Strauss et al., 2020). Furthermore, the fibrin matrix and leukocytes within PRF may contribute to modulating the inflammatory response and providing an antimicrobial effect (Drago et al., 2013), as suggested by our qualitative bacteriological and histological findings. However, this study has limitations. As a preclinical animal model, its direct translational applicability to human patients requires further validation through clinical trials. The mechanistic insights are based on histological outcomes; future studies incorporating quantitative molecular analyses (e.g., ELISA for VEGF, PDGF) would provide more direct evidence. Additionally, the inclusion of a PRF-treated non-seawater group would help delineate its specific effect on seawater-induced damage. Addressing these points will be the focus of our subsequent research.

### 4.5 Conclusion and clinical application value

In conclusion, autologous platelet-rich fibrin can promote wound healing, and its mechanism may be related to PRF promoting basal vascularization of wounds, and it can prevent infection of seawater immersion wounds, which has potential application for clinical treatment of seawater immersion wound treatment. In conclusion, autologous PRF significantly promotes the healing of seawater-immersed wounds in a rabbit model. The mechanism is multifactorial, involving the promotion of angiogenesis, reduction of inflammation, and potential inhibition of bacterial growth. PRF, with its facile preparation and autologous nature, presents a promising therapeutic strategy for the challenging clinical scenario of seawater immersion wounds.

## Data Availability

The original contributions presented in the study are included in the article/supplementary material, further inquiries can be directed to the corresponding authors.
